# Retrospective Evaluation of the Efficacy of Combined Antiviral Therapy Versus Monotherapy in High-Risk Hospitalized COVID-19 Patients

**DOI:** 10.7759/cureus.81903

**Published:** 2025-04-08

**Authors:** Hind Khalid Goresh, Aisha K Almutiri, Abeer O Hadari, Raghdaa M Alzain, Shatha K Altewajri, Manar Almutairi

**Affiliations:** 1 Clinical Pharmacy, Buraydah Colleges, Buraydah, SAU; 2 Pharmacy, Buraydah Colleges, Buraydah, SAU

**Keywords:** combination, covid-19, efficacy, favipiravir, hospitalization, monotherapy, mortality, remdesivir

## Abstract

The COVID-19 pandemic has led to a variety of clinical symptoms, ranging from mild respiratory problems to severe pneumonia and multi-organ failure. Remdesivir and favipiravir are approved to treat COVID-19 and are used as single agents in the Ministry of Health (MOH) protocol. However, there is limited research available on the effectiveness of their combined use. The aim of this cross-sectional study is to evaluate the efficacy and safety of remdesivir and favipiravir in reducing disease severity, hospitalization duration, and mortality rates among high-risk patients who have been hospitalized with COVID-19. A retrospective, cross-sectional study was conducted in adult patients who were treated with the MOH treatment protocol. The study period was from January 2021 to January 2022, and it included 47 patients treated with favipiravir or a combination of remdesivir and favipiravir. The study included patients with high-risk characteristics and excluded pediatric and pregnant patients. In a study of 47 hospitalized COVID-19 patients, all admitted cases presented with at least one COVID-19 symptom. The frequency of symptoms such as fever, cough, chest pain, shortness of breath, and fatigue was noted to be, respectively. The median duration of antiviral therapy was seven days (interquartile range (IQR): five to eight days), and one-third of study participants developed side effects due to antiviral administration. The proportions of renal and hepatic side effects were found to be comparable (25.5% and 21.3%, respectively). Furthermore, the frequency of overall symptom improvement on completion of antiviral therapy was found to be 70.2%. An improvement in oxygen saturation was found to be significant with combined antiviral therapy. A total of 10 death events were reported during the study period, yielding 22 poor outcomes per 1000 person-days. COVID-19 patients taking combined therapy had significantly longer survival times compared to those taking a single agent (15 and 23 days, P > 0.05, respectively). The cumulative probability of survival at the end of the study period among those receiving single and combined therapy was found to be 29% and 38%, respectively. The findings from the present study showed that combined remdesivir and favipiravir have a superior effect than single favipiravir medication in treating high-risk hospitalized COVID-19 patients. These results highlight the clinical importance of combined antiviral regimens in enhancing patient prognoses, reducing mortality rates and shortening hospital stays.

## Introduction

Coronaviruses are a group of respiratory viruses that cause diseases such as COVID-19, Middle East respiratory syndrome (MERS), and severe acute respiratory syndrome (SARS). SARS-CoV-2, which is a new coronavirus from the Coronavirus family, is believed to have originated in Wuhan, China. The leading hypothesis about its origin suggests that it became more pathogenic through a zoonotic event [1]. The rapid transmission of the infection was made possible by its ability to spread quickly, even through individuals who were asymptomatic. Furthermore, the limited capacity for testing, inefficient treatment methods, and the failure to implement early contact tracing and quarantine measures all contributed to the unprecedented global spread of the infection. The SARS-CoV-2 virus had an impressive ability to spread quickly among people worldwide through respiratory droplets, even when individuals did not exhibit any symptoms [1,2]. The COVID-19 outbreak quickly overwhelmed a significant number of hospitals and healthcare facilities globally, affecting almost every nation and demographic. Despite this unprecedented challenge, countries developed innovative treatments and created vaccines while also implementing measures to reduce the transmission of the virus between people. These measures included widespread quarantine and isolation, mandatory mask-wearing, and comprehensive stay-at-home orders in combination with the closure of businesses and gatherings [3]. In March 2020, the World Health Organization declared COVID-19 a global public health emergency. As of August 2021, there have been almost 200 million confirmed cases of SARS-CoV-2 worldwide and over 4.2 million deaths. This makes COVID-19 one of the deadliest epidemics in recorded human history [2]. This protocol offers evidence-based strategies for managing COVID-19 cases and includes a national treatment plan covering supportive care and medicines for all case levels. The Ministry of Health (MOH) monitors adherence to the protocol and provides guidelines for its implementation and reducing adverse effects [4]. Asymptomatic patients can end their self-isolation after 10 days, provided that they have been fever-free for at least three days and there has been a clinical improvement in symptoms. Pharmacotherapy is unnecessary for those who do not exhibit any symptoms, while mild to severe cases will be categorized based on clinical assessment and need urgent therapy for COVID-19 complications [5]. Remdesivir can cause infusion reactions, diarrhea, anemia, hyperglycemia, and elevated alanine aminotransferase (ALT)/serum glutamate pyruvate transaminase (SGPT) and aspartate aminotransferase (AST) levels on long-term treatment. It can affect kidney function, which might consequently lead to acute kidney injury (AKI) [6]. Favipiravir can cause liver impairment on long hospitalization, gastrointestinal effects, nausea, stomach discomfort, and rare instances of thrombocytopenia [7]. Studies show that antiviral drugs reduce mortality rates and shorten hospitalization durations. Single medicines like remdesivir or favipiravir are primarily used, and there is limited research on combined therapies, which are not yet approved [8,9].

## Materials and methods

Patients and settings

This study was a retrospective cross-sectional study performed on the medical registry, from January 2021 to January 2022, of governmental tertiary hospitals. Adult patients diagnosed with COVID-19 were identified after the study was ethically approved. This study consisted of two groups of patients using either favipiravir 1600 mg or a combination of remdesivir 100 mg and favipiravir 1600 mg once daily. The study gathered information on clinical characteristics and comorbidity. All patients who used combined therapy (remdesivir plus favipiravir) for managing the complications of COVID-19 were identified from the hospital registry in the period mentioned above. An equal number of patients using monotherapy (favipiravir) was identified based on match criteria. The study encompassed all adult patients who were hospitalized and had high-risk characteristics, such as chronic obstructive pulmonary disease (COPD), asthma, renal impairment, liver impairment, diabetes, and any cardiovascular illness. Pediatric patients, pregnant patients, non-hospitalized patients, and hospitalized patients without any risk factors were excluded from the study.

Study variables and outcomes

The primary outcome showed that the combination of remdesivir and favipiravir is more effective than using favipiravir alone in treating high-risk hospitalized COVID-19 patients, leading to reduced mortality rates and shorter hospital stays. COVID-19 patients taking combined therapy had significantly longer survival times compared to those taking a single agent (15 and 23 days, P > 0.05, respectively). On independent t-test, oxygen saturation improvement was found significantly correlated with combined anti-viral regimen (P = 0.04). However, paired t-test showed significant improvement in oxygen saturation level for combined and single antiviral group (P = 0.01 and less than 0.001, respectively).

Data analysis

Data analysis was undertaken using the IBM SPSS Statistics for Windows, Version 24 (Released 2017; IBM Corp., Armonk, New York, United States). Continuous variables were presented as mean and standard deviation (SD). Categorical variables were presented by percentages and frequencies. Hypothesis testing was carried out, as appropriate, to determine whether there was a statistically significant difference between the variables of interest using α = 0.05.

## Results

Demographic characteristics and outcomes

A total of 47 hospitalized COVID-19 patients were recruited for the study. The demographic data showed that half of the study participants were male (55%). Moreover, the age of the study participants ranges from 30 to 90 years (Table 1). The existence of comorbidities like hypertension, diabetes, renal impairment, and asthma or COPD was addressed to be 59.6% (n = 28), 48.9% (n = 23), 4.3% (n = 2) and 12.8% (n = 6), respectively (Table 1). The mean body weight of patients at admission was 75.9 ± 16.5 kg (mean ± SD).

**Table 1:** Demographic data of the study sample. COPD: Chronic obstructive pulmonary disease

Variable	n = 47	%
Age		
30-39	3	6.4
40-49	10	21.3
50-60	9	19.1
Above 60	25	53.2
Gender		
Male	26	55.3
Female	21	44.7
Comorbidity		
Hypertension	28	59.6
Diabetes mellitus	23	48.9
COPD/asthma	6	12.8
Obesity	13	27.7
Antiviral regimen		
Single	23	50
Combined	23	50
Outcome		
Alive	37	78.7
Death	10	21.3

Clinical characteristics and symptoms

All admitted cases presented with at least one COVID-19 symptom. The frequency of symptoms such as fever, cough, chest pain, shortness of breath, and fatigue was noted to be 70.2% (n = 33), 85.1% (n = 40), 12.8% (n = 6), 59.6% (n = 28) and 4.3% (n = 2), respectively (Table 2). The duration of antiviral therapy was noted to be a median of seven days. In addition, one-third (31.9%) of study participants developed side effects due to anti-viral administration. Proportions of renal and hepatic side effects were found to be comparable (25.5% and 21.3%, respectively). Furthermore, the frequency of overall symptom improvement on completion of antiviral therapy was found to be 70.2% (Table 2).

**Table 2:** Clinical characteristics of the study sample.

Symptoms on admission	n = 47	%
Fever	33	70.2
Cough	40	85.1
Chest pain	6	12.8
Shortness of breath	28	59.6
Fatigue	2	4.3
Current medications		
Antihypertensive	18	38.3
Anticoagulant	40	85.1
Antiplatelet	8	17
Antidiabetic	20	42.6
Bronchodilator	38	80.9
On admission, renal/cardiac/hepatic function		
Impaired renal function	12	25.5
Impaired liver function	16	34
Impaired cardiac function	8	17
Organ functions on completion of antiviral therapy		
Cardiac function improvement	9	19.1
Pulmonary function improvement	21	44.7
Overall function improvement	33	70.2

Association of clinical factors with COVID-19 treatment outcomes

On Chi-square and Fischer's exact test, impaired renal function, diabetes, and combined antiviral regimens were concluded to be significantly correlated with the treatment outcomes of COVID-19. Similarly, co-administration of antihypertensive and antiplatelet was significantly associated with the treatment outcomes. In contrast, fever, cough, chest pain, shortness of breath, and obesity were not associated with treatment outcome. Likewise, no adequate evidence to conclude a significant association with liver impairment, hypertension, asthma or COPD, antihypertensive, antidiabetic, and bronchodilator. Moreover, an insignificant difference was noted with regard to the adverse effect of antiviral regimens (P = 0.08). Oxygen saturation was measured on admission and at the end of treatment. On independent t-test, oxygen saturation improvement was found significantly correlated with combined antiviral regimen (P = 0.04). However, paired t-test showed significant improvement in oxygen saturation level for combined and single antiviral group (P = 0.01 and less than 0.001, respectively) (Table 3).

**Table 3:** Association of sociodemographic and clinical factors with treatment outcome. Statistical significance was determined using the Chi-square test, Fisher's exact test, and an independent t-test for categorical variables. The paired t-test was used to assess oxygen saturation improvement. * Statistically significant at p < 0.05. COPD: Chronic obstructive pulmonary disease

Variable	Alive (n)	Died (n)	P-value
Fever	25	8	0.45
Cough	32	8	0.61
Chest pain	5	1	0.77
Shortness of breath	23	5	0.49
Fatigue	1	1	0.38
Obesity	11	1	0.54
Impaired renal function	7	5	0.04*
Impaired liver function	12	4	0.7
Hypertension	20	8	0.11
Diabetes	15	8	0.02*
Asthma/COPD	5	1	0.76
Combined antiviral	5	6	0.002*
Antihypertensive	10	8	0.001*
Anticoagulant	31	9	0.45
Antiplatelet	3	5	<0.001*
Antidiabetic	13	7	0.013
Bronchodilator	28	10	0.74

The probability of survival of COVID-19 patients

**Figure 1:**
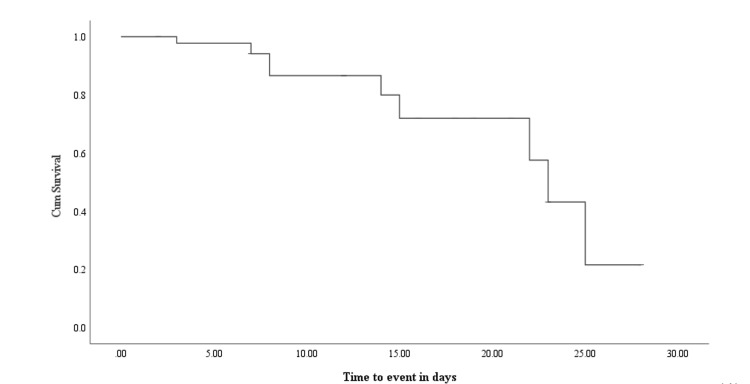
Kaplan-Meier curve showing the survival probability of COVID-19 patients from the start of treatment to the end of the follow-up period.

All study participants were followed in the hospital for a median of eight days with a total of 455 person-days (Figure 1).

**Figure 2:**
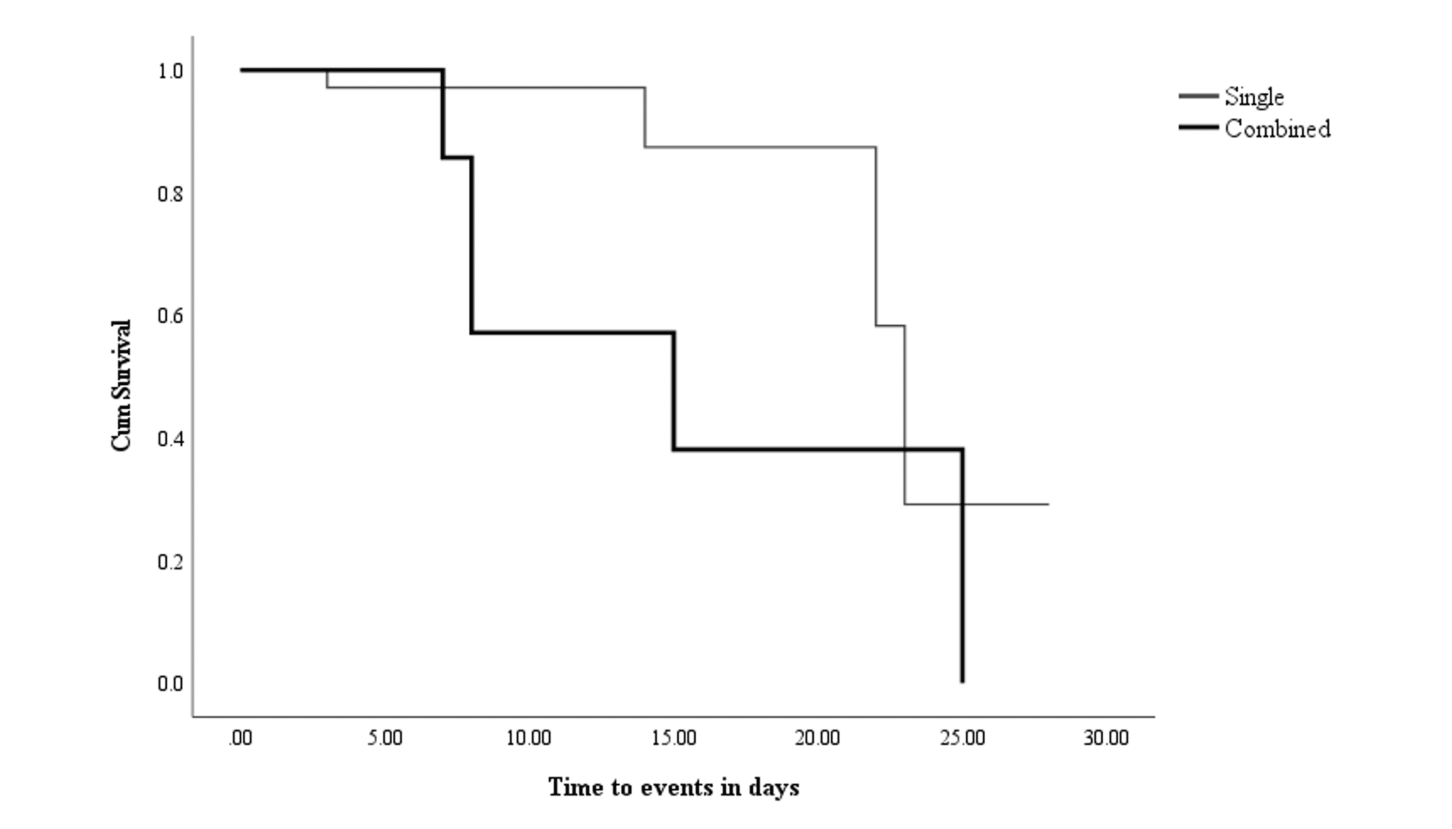
Kaplan-Meier survival probability curve comparing single and combined antiviral treatment groups.

A total of 10 death events were reported during the study period that yielded 22 poor outcomes per 1000 person-days. COVID-19 patients taking combined therapy had insignificantly shorter survival times compared with those taking single agents (15 and 23 months, P > 0.05, respectively). The cumulative probability of survival at the end of the study period among single and combined therapy was found to be 29% and 38%, respectively (Figure 2).

## Discussion

We believe that this study provides information regarding the efficacy and safety of single versus combined antiviral therapy among COVID-19 patients in Saudi Arabia to help evaluate treatment protocol in the country. Combination and single antiviral regimens are included in the treatment protocol of COVID-19 in the Kingdom.

The COVID-19 pandemic is a worldwide public health threat that yielded higher rates of mortality and affected the health of the elderly and people with comorbidities [10] and impacted morbidity and mortality. In our study, we found that 10 out of 47 (21.3%) developed poor outcomes (death). This value is lower than some studies conducted in Belgium (29.9%) [11] and China (28.3%) [12] and higher than that of Ethiopia (15.3%) [13]. These disparities could be attributed to the differences in the study sample, settings, study time, and study design.

Oxygen saturation improvement was found to be significantly associated with combined antiviral use; however, combined therapy was found to be significantly correlated with poor treatment outcomes (Table 3). Moreover, diabetes comorbidity, impaired renal function, antiplatelet use, and antihypertensives use were reported to be significantly associated with poor treatment outcomes (Table 3). To our knowledge, this is the first study in Saudi Arabia where combined versus single antiviral protocol is evaluated among hospitalized COVID-19 patients. Per protocol, in our study, our primary treatment outcomes were patient survival, whereas many secondary outcomes were evaluated to assess the efficacy and safety of the treatment regimen.

Improvement in clinical status, like oxygen saturation in our study, improved significantly with combined antiviral over monotherapy. This finding is consistent with related studies where combined antiviral therapy showed clinical improvement [14-16]. In contrast, Cai et al. reported that single antiviral showed chest improvement in computed tomography (CT) over combined therapy [17]. In addition, one study concluded that monotherapy had no benefit in improving clinical outcomes [18]. This contradiction could be attributed to the difference in the designs of these studies.

The adverse effects of antiviral regimens (combined versus single) showed an insignificant difference. A published article reported that combined antiviral therapy had fewer side effects compared to monotherapy [14,19], whereas Li et al. concluded that the adverse effect of combined therapy was higher compared with monotherapy [18]. On the other hand, Gao and his team addressed that the number of antiviral drugs had not predicted the occurrence of side effects [20].

Our study assessed the risk factors of poor outcomes (mortality) among hospitalized COVID-19 patients. Different published studies have assessed the existence of diabetes mellitus [21,22], COPD and asthma [23-25], HIV/AIDS, and chronic kidney disease [26]. Diabetes comorbidity was reported to be significantly associated with poor treatment outcomes in our study. Likewise, diabetes was reported to be a predictor of poor outcomes [21]. In addition, antihypertensive users predicted the occurrence of poor outcomes. Patients with cardiovascular disease like hypertension were found to be independent predictors of poor outcomes [21,27,28]. Impaired renal function was noted to be correlated with poor outcomes. Similarly, Tiwari et al. had concluded that renal impairment is an independent determinant of poor outcomes [29]. Furthermore, antiplatelet use was reported to be a risk factor of poor treatment outcome in our study, whereas a published retrospective cohort study reported that the use of anticoagulants had improved clinical outcomes [23].

In summary, diabetes comorbidity, impaired renal function, antiplatelet use, and antihypertensives use were concluded to be correlated with poor treatment outcomes. Consequently, addressing concerns with high blood glucose levels, renal impairment, and high blood pressure will be crucial in improving morbidity and mortality.

Study limitations

A cross-sectional study design was employed. Although this type of design is prone to selection bias, which is the main drawback, it was the most feasible option in this case. Additionally, the limited sample size may affect the study’s statistical power. Despite these limitations, we believe our findings are robust and can inform future research steps.

## Conclusions

The findings of this study indicate that the concurrent use of remdesivir and favipiravir demonstrates a greater efficacy compared to the administration of favipiravir alone in treating high-risk hospitalized patients with COVID-19. These results emphasize the significant clinical benefits of utilizing combined antiviral regimens, which enhance patient outcomes, reduce mortality rates, and shorten the duration of hospital stays. The implications of this research support the adoption of dual treatment strategies in clinical practice to optimize patient care in this critical context. Further studies with adequate sample sizes can be employed to highlight the efficacy and safety of different COVID-19 antiviral protocols. In addition, case-control or retrospective cohort designs can be adopted to address the predictors of poor treatment outcomes.
